# Impact of Artificial Intelligence-Based Autosegmentation of Organs at Risk in Low- and Middle-Income Countries

**DOI:** 10.1016/j.adro.2024.101638

**Published:** 2024-10-05

**Authors:** Solomon Kibudde, Awusi Kavuma, Yao Hao, Tianyu Zhao, Hiram Gay, Jacaranda Van Rheenen, Pavan Mukesh Jhaveri, Minjmaa Minjgee, Enkhsetseg Vanchinbazar, Urdenekhuu Nansalmaa, Baozhou Sun

**Affiliations:** a Division of Radiation Oncology, Uganda Cancer Institute, Kampala, Uganda; b Division of Radiation Oncology, Washington University in St. Louis, St. Louis, Missouri; c Department of Radiation Oncology, Baylor College of Medicine, Houston, Texas; d Department of Radiation Oncology, National Cancer Center of Mongolia, Ulaanbaatar, Mongolia

## Abstract

**Purpose:**

Radiation therapy (RT) processes require significant human resources and expertise, creating a barrier to rapid RT deployment in low- and middle-income countries (LMICs). Accurate segmentation of tumor targets and organs at risk (OARs) is crucial for optimal RT. This study assessed the impact of artificial intelligence (AI)-based autosegmentation of OARs in 2 LMICs.

**Methods and Materials:**

Ten patients, comprising 5 head and neck (HN) cancer patients and 5 prostate cancer patients, were randomly selected. Planning computed tomography images were subjected to autosegmentation using an Food and Drug Administration-approved AI software tool and manual segmentation by experienced radiation oncologists from 2 LMIC RT clinics. The control data, obtained from a large academic institution in the United States, consisted of contours obtained by an experienced radiation oncologist. The segmentation time, DICE similarity coefficient (DSC), Hausdorff distance, and mean surface distance were evaluated.

**Results:**

AI significantly reduced segmentation time, averaging 2 minutes per patient, compared with 57 to 84 minutes for manual contouring in LMICs. Compared with the control data, the AI pelvic contours provided better agreement than did the LMIC manual contours (mean DSC of 0.834 vs 0.807 in LMIC1 and 0.844 vs 0.801 in LMIC2). For HN contours, AI provided better agreement for the majority of OAR contours than manual contours in LMIC1 (mean DSC: 0.823 vs 0.821) or LMIC2 (mean DSC: 0.792 vs 0.748). Neither the AI nor LMIC manual contours had good agreement with the control data (DSC < 0.600) for the optic nerves, chiasm, and cochlea.

**Conclusions:**

AI-based autosegmentation generates OAR contours of comparable quality to manual segmentation for both pelvic and HN cancer patients in LMICs, with substantial time savings.

## Introduction

By 2040, the global landscape of oncology is projected to surge, with an estimated 28.4 million new cancer cases, which is a 47% increase from the 19.3 million cases recorded in 2020.^1^ Alarmingly, although 3-quarters of this growing cancer burden will be shouldered by low- and middle-income countries (LMICs), these countries are equipped with less than 5% of the world's health resources dedicated to cancer treatment.^2^ This stark resource disparity has direct ramifications for treatment outcomes, resulting in significantly poorer results in LMICs for comparable cancer stages and types observed in high-income nations.

Contributing to this outcome discrepancy is not only restricted access to preventive measures and early detection initiatives but also the acute scarcity of expert personnel and specialized cancer treatment equipment.^3^ It is anticipated that more than half of cancer patients globally will require radiation therapy at some juncture during treatment, either for curative intent or disease control. However, the provision of radiation therapy in LMICs is fraught with significant challenges.

Although progress has been made to improve radiation therapy access in LMICs, data from March 2020 revealed that only 52% of the 54 nations in Africa had facilities equipped with external beam radiation therapy.^4^ Moreover, a mere 39% boasted brachytherapy capabilities, with none meeting the burgeoning demand.^4^ Beyond the imperative for infrastructural and technological investments, the dearth of qualified professionals in LMICs to steer these initiatives remains a pressing concern. Comprehensive training for a radiation therapy unit is not only time-intensive but also financially taxing. Projections indicate an impending demand for 12,960 radiation oncologists, 6480 medical physicists, 3240 dosimetrists, and 20,520 radiation therapists.^5^ The fiscal aspects of such training are less conspicuous in nations with established radiation therapy infrastructure and available training initiatives.^5^

Radiation therapy, by nature, is a multifaceted process. It involves a gamut of stages ranging from initial consultations and simulations to the crucial phases of target delineation, planning, and quality assurance, culminating in treatment delivery, response evaluations, and subsequent follow-ups. This intricate sequence necessitates collaborative effort from an ensemble of professionals, including radiation oncologists, physicists, therapists, dosimetrists, nurses, and ancillary staff. Under typical circumstances, the interval spanning simulation to treatment initiation extends to 2 weeks or more, a period during which the oncologist meticulously contours the tumor and the organs at risk (OARs).

Emerging technologies, specifically artificial intelligence (AI), hold promise for expediting some of these processes. The adoption of AI for functions such as contouring and autoplanning is witnessing increasing acceptance in contemporary oncology.^6^^,^^7^ Many affluent countries are already harnessing AI-powered contouring tools, reaping benefits such as time conservation, minimized interobserver discrepancies,^8^^,^^9^ and, notably, a reduced lag between simulation and treatment onset.

Systemic challenges, including staffing shortages in LMIC-based radiation therapy centers that consequentially lead to inconsistent patient outcomes, can potentially be mitigated with AI integration. Given the labor-intensive nature of the radiation therapy workflow, characterized by multiple manual interventions from a team of specialists,^10^ the implementation of AI could be transformative. Decreasing workloads would allow the scarce medical workforce in LMICs to direct their efforts toward enhanced patient interaction and care. In this paper, we report the implementation of AI-based autosegmentation and its impact on the radiation therapy workflow in 2 distinct LMICs: Uganda Cancer Institute and National Cancer Center of Mongolia.

## Materials and Methods

### LMIC settings

#### Uganda Cancer Institute (UCI) in LMIC1

This institution is the national cancer referral, receiving approximately 7000 new cases of 34,000 diagnosed annually. The only radiation therapy facility located in the country treats approximately 2500 new patients annually (less than 8% of cancer patients are treated with radiation therapy). Since its establishment in 1995 with support from the International Atomic Energy Agency (IAEA), the radiation therapy department offered conventional 2-dimension (2D) radiation therapy until March 2021, when only Linac was commissioned, paving the way for a transition toward 3-dimension (3D) conformal radiation therapy and intensity modulated radiation therapy (IMRT)/volumetric-modulated arc therapy (VMAT) with support from Washington University in St Louis in the United States and IAEA. The center has 2 Cobalt-60 units, 3 TrueBeam Linacs, and 2 high-dose-rate brachytherapy units in clinical use. Each of the Truebeam Linacs has an estimated 50 patients receiving daily treatment. The department has 4 radiation oncologists, 10 radiation therapists, and 4 medical physicists. All the contours of the radiation oncologists, including OARs and targets, were drawn in Eclipse (Varian Medical Systems).

#### National Cancer Center of Mongolia (NCCM) in LMIC2

This institution is the only radiation therapy facility in Mongolia, with 800 referrals of 6000 new cancer cases reported in 2020. Only approximately 13% of cancer patients are treated with radiation therapy. Since 1995, the IAEA has implemented several projects to improve the quality, scope, and scale of radiation therapy services in LMICs2. The department has 2 Linacs, commissioned in 2019. A TrueBeam Linac is being installed and planned to treat patients in early 2024. Currently, there are 10 radiation oncologists, 10 radiation therapists and 4 medical physicists at the National Cancer Center of Mongolia. A previous study shows that the NCCM has recently started IMRT treatments with support from the IAEA and Washington University in St Louis (UWSTL).^11^ With the capital investment and additional linacs being commissioned, both LMIC1 and LMIC2 are expected to treat more patients with radiation therapy. The throughput will be limited by the number of staff and training needed.

### Design

This study followed a cross-sectional design, encompassing a sample of 10 patients, comprising 5 individuals with head and neck (HN) cancer and 5 with prostate cancer. The cross-sectional study was conducted over a period from January 2023 to February 2023. During this time, both LMICs involved in the study were in a transition period from 2D radiation therapy techniques to more advanced IMRT technique.

### Computed tomography simulation and contouring for control data

The computed tomography (CT) scan image data sets used for radiation therapy planning (with patient anonymity ensured) were generously provided by the UWSTL in the United States. The selection of these CT scans followed a random sampling methodology from the pool of patients previously treated at UWSTL in the United States. For HN cancers, CT scans were acquired with a slice thickness of 2 mm, while for prostate cancer, the slice thickness was 3mm. Contours were drawn by experienced radiation oncologists and dosimetrists at a prominent academic institution in the United States. These contours served as the control data and “ground truth.” The same planning CT image data sets were used for the 10 patients who underwent the following 2 different segmentation procedures:

1) Automated segmentation using a Food and Drug Administration (FDA)-approved AI software tool, and
2) Contours were drawn by proficient radiation oncologists at LMIC1 and LMIC2.

### Manual contouring process at LMIC Institutions

The same planning CT data sets sampled from high-income country (HIC) clinic in United States were uploaded to Eclipse Treatment Planning System (TPS) for contouring. In LMIC1, the study used the Eclipse TPS version 16.1, whereas in LMIC2, the TPS used was Eclipse version 15.7. Experienced radiation oncologists at LMICs performed manual contouring following standard protocols. For the HN, the OAR were contoured according to international consensus guidelines.^12^ For prostate cancer patients, contouring of OAR was performed according to the Radiation Therapy Oncology Group Consensus Panel Atlas.^13^ For prostate cancer, the contours delineated were: the prostate, the seminal vesicles, the urinary bladder, the rectum, the penile bulb, and both femoral heads. The inclusion of sigmoid and bowel contours was not considered in this study because of variations in contouring approach among the 3 institutions. On the other hand, for HN cancer, the delineations encompassed the brain, brainstem, bilateral eyes, lens, optic nerves, cochlea, parotids, optic chiasm, spinal cord, oral cavity, and mandible. The existing contouring tools within the Eclipse TPS used in LMIC1 and LMIC2 were leveraged to varying extents. These tools included interpolation, adaptive paint, flooding, and autosegmentation.

It should be noted that the control contours were based solely on CT scans to ensure consistency in comparison.

### Autosegmentation workflow

To enhance the capabilities of radiation oncologists at UCI and NCCM institutions in low-income countries, CARINA-AI facilitates training sessions. Throughout the study, the INTContour platform by CARINA-AI was employed for autosegmentation, adhering to strict anonymized usage protocols. INTContour (CARICA AI), a web-based tool using deep learning algorithms for autosegmentation,^14^ was employed to automatically generate contours for corresponding anatomic sites for each of the 10 patients. The CARINA-AI system was trained using a diverse data set from HIC settings encompassing a wide range of racial, sex, and age groups to account for anatomic differences. It contains built-in models that enable incremental learning based on user-provided data.

The workflow involves the following steps:

1. *Data upload*: CT simulation data are uploaded to the CARINA-AI web-based platform by the radiation therapy staff.
2. *Autosegmentation*: The uploaded data are processed by the AI system to generate contours for the anatomic sites. This step takes approximately 2 minutes per case.
3. *Review and approval:* The AI-generated contours are reviewed and approved by radiation oncologists to ensure accuracy and clinical relevance.
4. *Data transfer*: The approved contours are then downloaded and imported back into the Eclipse planning system for further treatment planning.

The overall time required for human intervention between the CT scan and the start of planning includes data upload, review, and approval, which is estimated to be around 10 minutes.

### Measurement of efficiency

A standardized model (generic models by CARINA-AI), initially trained on patients from high-income countries, was employed to assess the efficiency of autosegmentation in 10 patients previously treated at the UWSTL institution in the United States. The planning CT scans were uploaded to the web-based segmentation tool. Efficiency was assessed by comparing the duration in minutes required for autosegmentation with that required for manual segmentation performed by experienced radiation oncologists from 2 institutions in the LMICs.

### Measurement of quality

We used the 2 most commonly used evaluation metrics, the Dice similarity coefficient (DSC) and Hausdorff distance (HD), to measure the spatial overlap between the test data (AI-generated contours or LMIC contours) and control data^15^:

$$\text{DSC}\;=\frac{2\;\times \left|True\cap Test\right|}{\left|True\right|\;+\;\left|Test\right|},$$

where |True| and |Test| are the numbers of pixels in the control data and test data, respectively.

The average HD is the average distance of a set X to the nearest point in the other set Y, given as

$${\vec{d}}_{H,avg}\left(X,Y\right)=\frac{1}{\left|X\right|}\sum_{x\in \left|X\right|}min_{y\in \left|Y\right|}d\left(x,y\right)$$

The mean surface distance (MSD) is then defined as the average of the 2 directed average Hausdorff measures^5^:

$$\text{MSD}=\frac{{\vec{d}}_{H,avg}\left(X,Y\right)+{\vec{d}}_{H,avg}\left(Y,X\right)}{2}$$

The HD95^16^ is based on the calculation of the 95th percentile of the distances between boundary points in X and Y:

$$d_{HD95}\left(X,Y\right)=max_{95\%}\left(d_{XY,\;}d_{YX\;}\right)=max_{95\%}\left[min_{y\in Y}d\left(x,y\right),max_{y\in Y}min_{x\in X}d\left(x,y\right)\right],$$

where x represents a point on test contour X, y is a point on the ground truth contour Y, and d(x,y) is the Euclidean distance between point x and point y.

The DSC, MSD, and HD95 between the AI-generated data and control data and between the LMIC contours and control data were compared to determine the similarity of the different contour data sets.

## Results

### Segmentation efficiency

The average processing time for contouring OAR volumes was ∼2 minutes per case for the AI-based model for patients with prostate cancer or HN cancer, compared with 62.8 minutes (range, 57-73 mins) per case for manual contouring for patients with prostate cancer and 50.75 minutes (range, 50-52 mins) per case for manual contouring for patients with HN cancer in the UCI in LMIC1 and 84 minutes per manual contour for patients with HN cancer or prostate cancer in the NCCM in LMIC2.

### Prostate cancer patients: evaluation of segmentation accuracy

**Figure 1:**
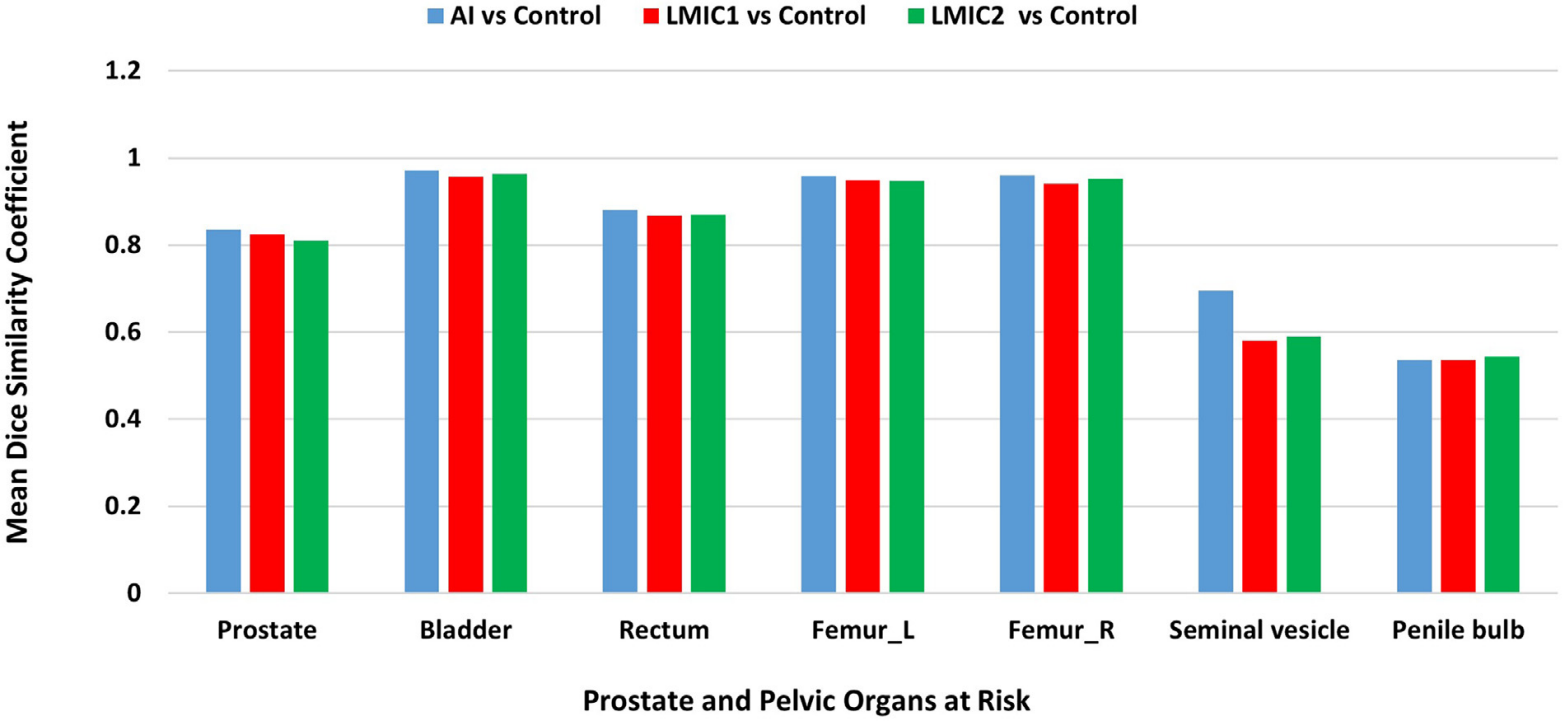
DICE similarity coefficients for pelvic OARs between control data and AI, LMIC1, and LMIC2. *Abbreviations:* LMIC = low- and middle-income country; OAR = organ at risk.

AI pelvic contours provided slightly better agreement than manual contours from either of the LMIC institutions for all 5 OARs and the prostate (Fig. 1). The analysis of the OAR contour quality showed that the bladder exhibited the highest mean DSC of 0.971, whereas the penile bulb demonstrated the lowest mean DSC of 0.536, followed by the seminal vesicles.

A comparison of the accuracy of prostate cancer OAR contours between the UWSTL in the United States and those generated using AI, as well as between the 2 LMIC institutions and the control, is provided (Table 1). There was high agreement in the DSC for the prostate, bladder, rectum, and femur heads between AI-generated contours and manual contours from LMIC institutions. The least DSC was observed with contours for the seminal vesicles, either between the AI and control or between the control and LMIC institutions. The HD95 was highest for the seminal vesicle at 9.599 mm between LMIC1 and the control, compared with 4.743 mm between AI and the control. There was a marked difference in the HD95 between the contours for the rectum generated by the AI (4.349 mm) and those generated by either the LMIC1 (5.110 mm) or the LMIC2 (4.594 mm). The least variation was observed with contours for the urinary bladder and prostate gland. The MSD, which refers to how much, on average, the surface varies between the manual and AI segmentations (in mm),^17^ values demonstrate reasonable proximity between contours, with the lowest MSD values observed with the comparison of urinary bladder contours between LMIC2 and the control (0.783 mm). The widest MSD is observed with contours for the seminal vesicle between LMIC1 and the control (3.313 mm).

**Table 1:** DSC, MSD, and Hausdorff distance (HD95) of prostate cancer OAR contours

OAR	Parameter	AI vs control	LMIC1 vs control	LMIC2 vs control
Prostate	DSC	0.836	0.824	0.811
	MSD (mm)	1.991	2.037	1.942
	HD95 (mm)	5.035	5.604	5.591
Bladder	DSC (mm)	0.971	0.958	0.963
	MSD (mm)	0.784	0.863	0.783
	HD95 (mm)	2.094	2.227	2.299
Rectum	DSC	0.880	0.867	0.869
	MSD (mm)	1.466	1.617	1.413
	HD95 (mm)	4.349	5.110	4.594
Femur_L	DSC	0.960	0.949	0.948
	MSD (mm)	0.725	0.878	0.927
	HD95 (mm)	1.961	3.719	3.931
Femur_R	DSC	0.959	0.941	0.952
	MSD (mm)	0.745	1.034	0.863
	HD95 (mm)	1.930	5.154	3.882
Seminal vesicle	DSC	0.629	0.580	0.589
	MSD (mm)	1.662	3.313	2.623
	HD95 (mm)	4.743	9.599	7.301
Penile bulb	DSC	0.536	0.535	0.544
	MSD (mm)	2.332	2.315	2.302
	HD95 (mm)	5.422	5.424	5.427

*Abbreviations:* DSC = DICE similarity coefficient; LMIC = low- and middle-income country; MSD = mean surface distance; OAR = organ at risk.

The comparison of contour accuracy between experts from the UWSTL institution of the HIC and UCI and NCCM institutions of the LMIC revealed variation in the MSD for pelvic OAR contours between the 2 LMIC institutions. The variation in the MSD values for manual contours from LMIC institutions was least for the urinary bladder (0.863 mm for LMIC1 compared to 0.783 mm for LMIC2), followed by the left femur (0.878 mm for LMIC1 compared to 0.927 mm for LMIC2). The largest MSD variation between the contours for the UWSTL institution in the United States and those for the 2 LMIC institutions was observed for the seminal vesicles (3.313 mm for LMIC1 and 2.623 mm for LMIC2; Table 1).

**Figure 2:**
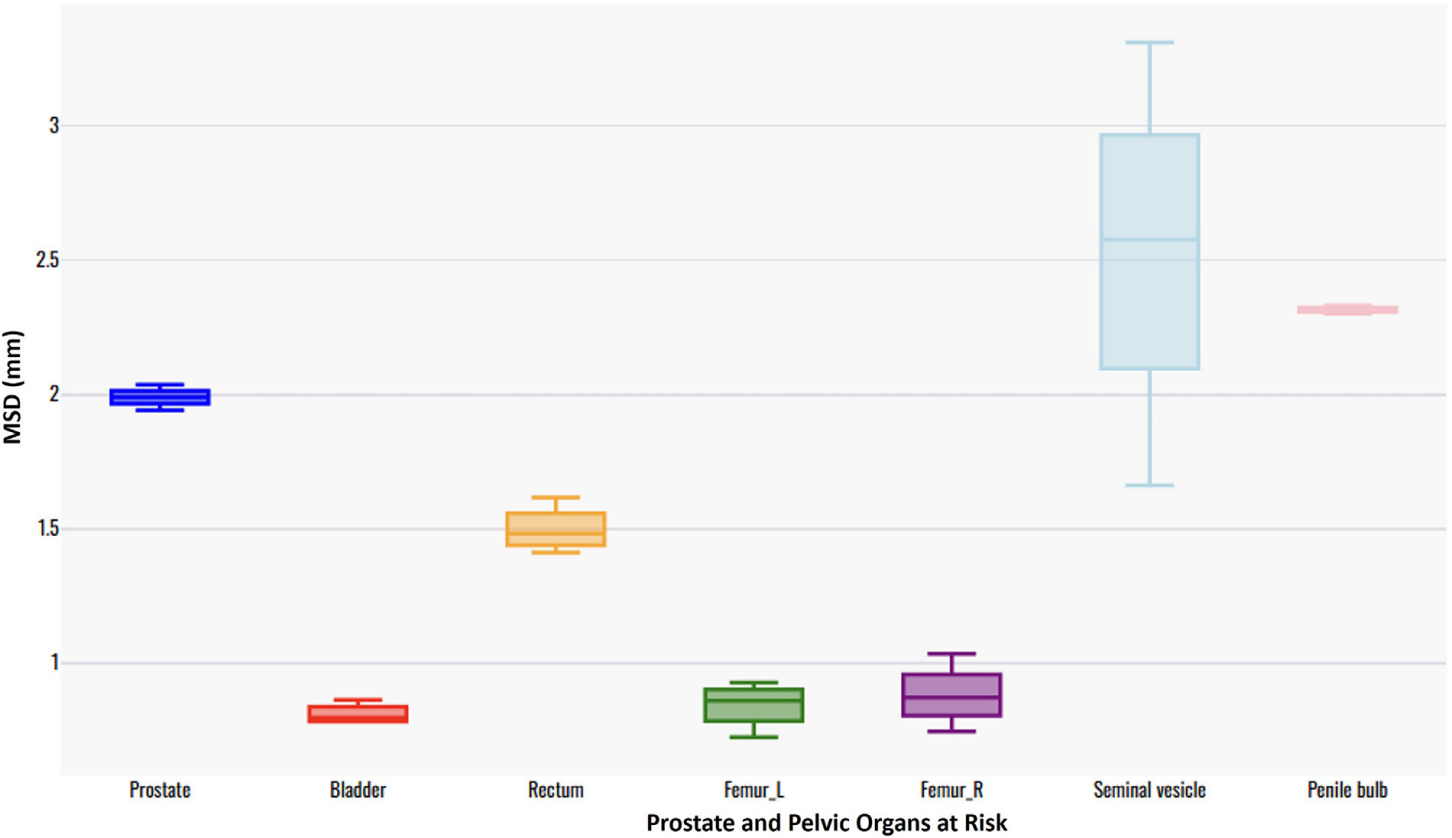
Box plot showing the mean surface distance (MSD) for pelvic OAR contours for the 3 groups (LMIC1 vs control, AI vs control, and LMIC2 vs control). *Abbreviations:* LMIC = low- and middle-income country; OAR = organ at risk.

Figure 2 shows the MSD for contours for the prostate, urinary bladder, rectum, femurs, and seminal vesicles for patients with prostate cancer, contoured by an experienced Radiation Oncologist (RO) from the UWSTL institution in the HIC, compared with manual contours by an experienced RO from 2 UCI and NCCM institutions in the LMIC. In general, the 2 sets of contours exhibit good agreement, as indicated by relatively low MSD values for the urinary bladder and femurs. Notably, the MSD for the prostate from all the contours was 2 mm, whereas that for the rectum was approximately 1.5 mm. The largest variation in the MSD was observed with the contours of the seminal vesicles.

### HN cancer: evaluation of segmentation accuracy

**Figure 3:**
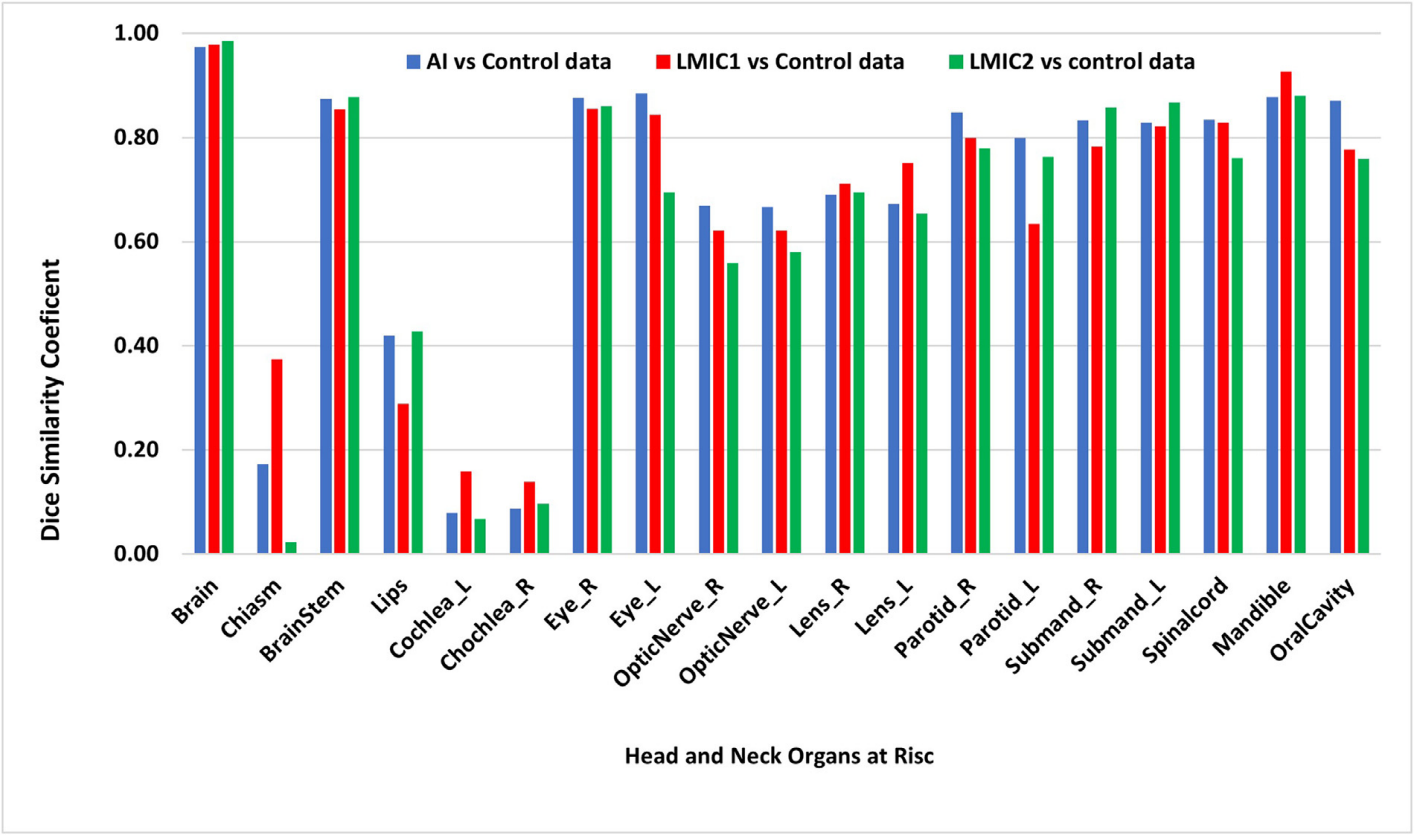
DICE similarity coefficients for HN OAR contours between LMIC1, LMIC2, control, and AI. *Abbreviations:* LMIC = low- and middle-income country; OAR = organ at risk.

Figure 3 shows DSC scores for the contours of the brain, brainstem, cochlea, eyes, lens, larynx, lips, mandible, optic nerves, parotids, and spinal cord for the 5 patients with HN cancer generated from UWSTL institution in HIC and 2 LMIC institutions compared with AI contours. Overall, there is a high level of agreement between the contours of experts at the UWSTL institution in HIC and AI-generated contours, as indicated by the DSC scores, averaging close to or above 0.8 for most organs. However, DSC scores were low (less than 0.5) for the chiasm, lips, and cochleae. Notably, the DSC scores were greater not only for the chiasm and cochlear contours from LMIC1 but also for the mandible and lens. Contours from LMIC2 showed the highest DSC scores for the submandibular gland, brain, lips, and brainstem. AI contours showed the highest DSC scores for contours for the oral cavity, spinal cord, parotids, optic nerves, and eyes.

**Figure 4:**
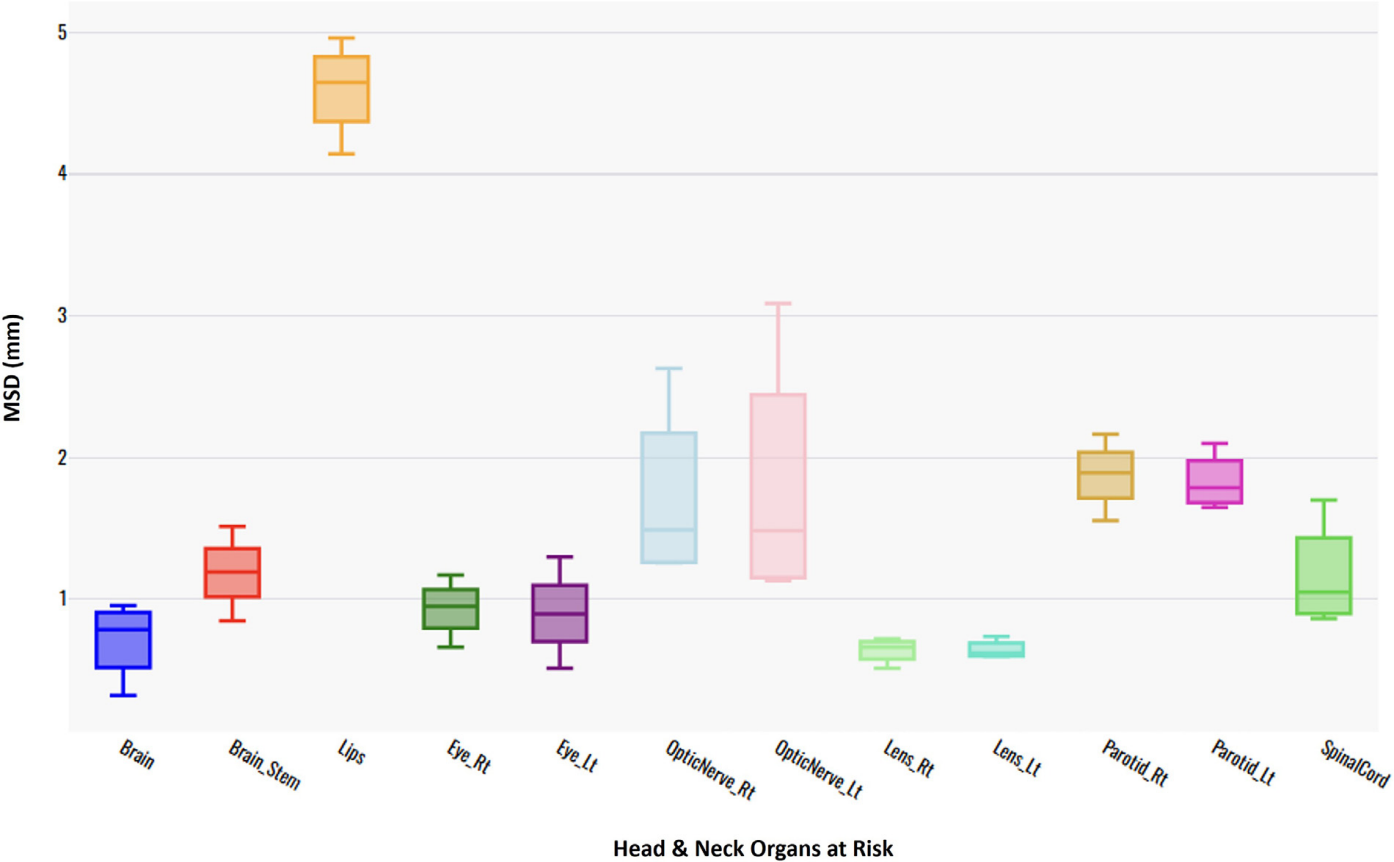
Box plot showing the mean surface distance (MSD) for HN OAR contours for the 3 groups (LMIC1 vs control, AI vs control, and LMIC2 vs control). *Abbreviations:* LMIC = low- and middle-income country; OAR = organ at risk.

Figure 4 shows the MSD for contours for the brain, brainstem, cochlear, eyes, lens, larynx, lips, mandible, optic nerves, parotids, and spinal cord for patients with HN cancer, contoured by an experienced RO from the UWSTL institution in the HIC, compared with manual contours from 2 UCI and NCCM institutions in the LMIC. In general, the 2 sets of contours exhibit good agreement, as indicated by relatively low MSD values for most organs. The least variation in the MSD was observed for the lens, brain, eyes, and spinal cord. The largest variation in the MSD values was observed for the lips, right parotid, and optic nerves.

A comparison of the DSC, MSD, and HD95 metrics for HN OAR contours generated by a radiation oncologist from the UWSTL institution in a HIC, 2 institutions in LMICs, and AI-generated contours is shown (Table 2). There was high agreement between AI-generated and manually generated contours from the 2 LMICs for the brain, brainstem, eyes, parotid, and spinal cord, with DSC scores >0.8. There was low agreement between AI-generated and manually generated contours from the 2 LMICs for the lips, optic nerves, and lens, with DSC values <0.7. The lowest DSC scores were observed for the lips. The variation in the HD95 was greatest for the lips at 11.432 mm between the AI treatment and the control, 10.154 mm between the LMIC1 treatment and the control, and 15.484 mm between the LMIC2 treatment and the control. Furthermore, the HD95 for the right optic nerve contours was 4.831 mm for the AI group vs the control group and 10.848 mm for the LMIC2 group vs the control group. Significant differences in HD95 were observed, with spinal cord contours of 2.617 mm for the AI group vs the control group and 6.463 mm for the LMIC2 group vs the control group. The least variation in HD95 was observed with the lens, with an HD95 of 1.926 mm for the AI group vs the control group, 2.304 mm for the LMIC1 group vs the control group, and 1.647 mm for the LMIC2 group vs the control group.

**Table 2:** DSC, MSD, and Hausdorff distance (HD95) of head and neck cancer OAR

OAR	Parameter	AI vs control	LMIC1 vs control	LMIC2 vs control
Brain	DSC	0.973	0.983	0.985
	MSD (mm)	0.859	0.955	0.322
	HD95 (mm)	3.578	3.763	1.179
Brainstem	DSC	0.866	0.852	0.878
	MSD (mm)	1.514	1.201	0.846
	HD95 (mm)	4.348	3.758	2.703
Lips	DSC	0.486	0.360	0.428
	MSD (mm)	4.963	4.696	4.143
	HD95 (mm)	11.432	10.154	15.484
Eye_R	DSC	0.867	0.836	0.860
	MSD (mm)	1.170	0.968	0.661
	HD95 (mm)	3.569	3.195	1.532
Eye_L	DSC	0.885	0.834	0.873
	MSD (mm)	0.892	1.297	0.611
	HD95 (mm)	2.732	3.512	1.647
Optic_Nerve_R	DSC	0.655	0.591	0.559
	MSD (mm)	1.257	1.263	2.630
	HD95 (mm)	4.831	5.476	10.848
Optic_Nerve_L	DSC	0.656	0.621	0.559
	MSD (mm)	1.609	1.143	2.630
	HD95 (mm)	5.079	4.240	13.772
Lens_R	DSC	0.671	0.683	0.695
	MSD (mm)	0.721	0.681	0.513
	HD95 (mm)	1.926	2.304	1.647
Lens_L	DSC	0.653	0.731	0.695
	MSD (mm)	0.704	0.514	1.647
	HD95 (mm)	1.703	1.672	2.884
Parotid_R	DSC	0.847	0.800	0.779
	MSD (mm)	2.166	1.554	1.907
	HD95 (mm)	6.363	4.823	6.624
Parotid_L	DSC	0.809	0.788	0.763
	MSD (mm)	1.621	1.909	1.714
	HD95 (mm)	5.430	7.283	6.042
Spinal cord	DSC	0.837	0.821	0.761
	MSD (mm)	0.932	0.864	1.700
	HD95 (mm)	2.617	2.217	6.463
Oral_Cavity	DSC	0.870	0.792	0.760
	MSD (mm)	2.801	2.558	3.211
	HD95 (mm)	9.663	7.521	10.712

*Abbreviations:* DSC = DICE similarity coefficient; LMIC = low- and middle-income country; MSD = mean surface distance; OAR = organ at risk.

## Discussion

The findings of this study highlight the potential benefits of incorporating AI-based autosegmentation of OARs in LMICs. Compared with manual contouring, autosegmentation demonstrated similar quality to human contouring from LMICs but achieved significant gains in efficiency for patients with prostate cancer and HN cancer. The average processing time for AI-based contouring was notably shorter, at 2 minutes per case, compared to manual contouring times ranging from 50.75 to 84 minutes per case in LMIC institutions. This work contributes to the existing body of literature indicating that autosegmentation has significant efficiency gains compared to manual contouring for patients with prostate cancer and HN cancer.^18^, ^19^, ^20^ Numerous studies have focused on models trained using patients from HICs. However, in this research, we conducted a comparative analysis of contours using identical planning CT data across experts from both low- and high-income countries (LMICs and HICs) and AI algorithms. Furthermore, the duration of automated segmentation was notably shorter than that in prior reports, taking only 2 minutes instead of the previously reported 3 minutes for both prostate cancer and HN cancer patients^21^ and 2.3 minutes (range, 1.2-8) for a variety of tumor sites.^22^

**Figure 5:**
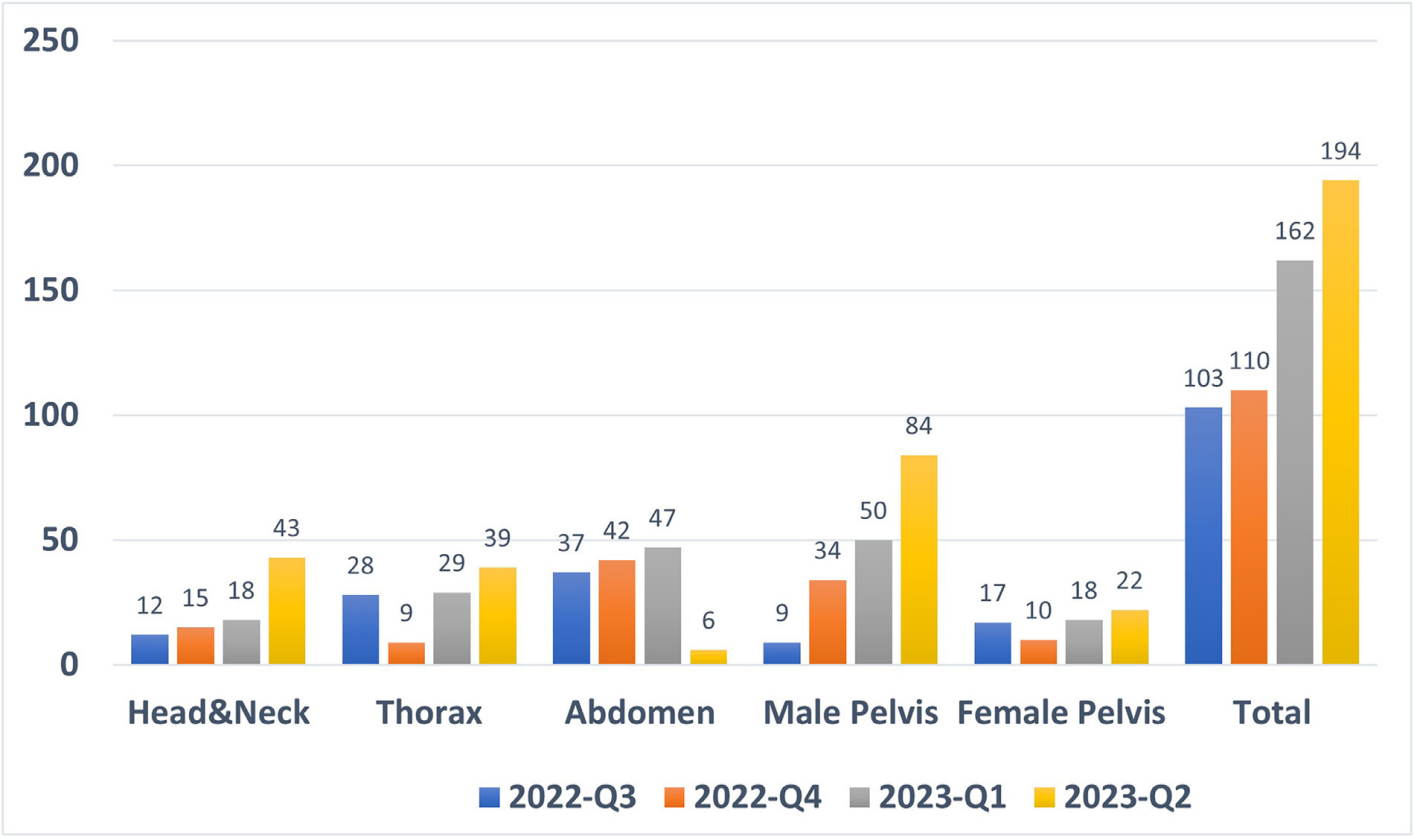
Autosegmentation of LMIC1 performed at the XXX institution over 1 year. *Abbreviations:* LMIC = low- and middle-income country; OAR = organ at risk.

Approximately 1000 patients receive conformal radiation therapy at the UCI each year (Fig. 5). Autosegmentation reduced the manual contouring time (∼60 minutes per case) to 2 minutes, resulting in a savings of ∼1000 hours annually. Such efficiency gains hold significant importance for a resource-constrained clinic, making a substantial impact.^6^ Physicians can focus their efforts on seeing more patients or other critical tasks.

AI technology has emerged as a promising solution for enhancing the standardization, efficiency, and quality of radiation therapy workflows. By automating critical steps, more accurate and safer radiation treatments can be achieved. In HICs, the adoption of AI-based autosegmentation and knowledge-based planning has already begun to reshape radiation therapy workflows, enhancing efficiency. Several AI-based autosegmentation tools have received approval from the United States FDA. However, when considering the implementation of such AI-based solutions in LMICs, challenges abound. These limitations include a scarcity of data for AI model training and validation, an absence of established AI-focused educational curricula in radiation therapy, limited numbers of experts trained in AI-driven radiation therapy, limited funding, and a general lack of AI infrastructure and resources.^23^

In this study, we introduced an FDA-approved software tool, Carina-AI, to 2 countries with markedly limited resources. The AI models were trained using patient data from HICs and were well-validated. It should be noted that AI can enhance the accuracy and consistency of contouring, but it cannot replace the knowledge and clinical judgment of radiation oncologists, physicists, or radiation therapists. Therefore, all AI-generated contours were reviewed and approved by radiation oncologists, who are the ultimate gatekeepers of treatment quality—a practice consistent with that in HICs. The utilization of a web-based software tool effectively mitigates the need for extensive IT support from local clinics in LMICs. Moreover, this tool facilitates faster peer review by providing a platform that allows the secure distribution of planning data sets between radiation centers for external evaluation and feedback. However, transferring CT data to the cloud poses its own set of challenges, especially given the often-limited internet speeds in LMICs. At LMIC1, we delegated data transfer tasks, including both exporting and importing, to radiation therapists who have undergone basic training. This delegation was necessary because of the limited availability of IT support staff. The involvement of therapists in this process ensured efficient data management and minimized delays in the workflow. Overtime, medical physicists have embraced this task.

There were several limitations and challenges in this study:

1) The control data used in this study originated primarily from radiation oncologists and dosimetrists at a single institution. Although an effort has been made to maintain objectivity and broader applicability by incorporating a review by a secondary radiation oncologist from a different HIC, it may not be sufficient to serve as a ground truth for contours. Additionally, it is worth noting that most "expert" centers define their contours on MRI and additional imaging. This reliance on advanced imaging modalities, which are not always available in LMICs, may contribute to differences in contour accuracy and size.
2) The AI-based autosegmentation tool employed in this study demonstrated certain limitations in accurately segmenting smaller organs, notably the optic nerves and cochlea, among others. It is vital to recognize that precise delineation of these smaller structures is crucial given their sensitivity and the potential consequences of missegmentation during radiation therapy. This limitation has been reported in several studies.^24^^,^^25^ This limitation exists not only for AI-based contours but also for manual contouring from LMICs, which exhibited similar discrepancies when compared with the control data. This observation underscores potential variability in contouring methodologies and practices across different regions and institutions. Additionally, although the parameters used for contour quality comparison are fairly satisfactory, it should be noted that the HD95 metric, being a linear measurement, has limitations in volumetric assessment. This limitation has been highlighted in other similar studies, indicating the HD95 may not fully capture the complexities of contour accuracy and volume differences.^26^^,^^27^
3) It is important to note that the study was conducted during a transition period from 2D to IMRT in both LMICs, spanning from January 2023 to February 2024. At both participating LMIC institutions, this transition period reflects a strain on the limited personnel by the addition of tasks such as target and organ at risk segmentation during IMRT planning. Notably, these technologies facilitated the deployment of advanced radiation therapy techniques in both LMIC institutions.

In the current study, we excluded contours for the sigmoid colon and bowel bag, because of significant variations in contouring approach among the 3 institutions. Future studies could explore these additional structures to provide a more comprehensive analysis.

To the best of our knowledge, this study represents a pioneering exploration of the potential impact of AI on radiation therapy in LMICs. Although there are undeniable challenges and limitations associated with the integration of AI into radiation therapy workflows in these regions, the benefits are substantial. The implementation of AI has the potential to save significant resources, address the pressing issue of workforce shortages in radiation therapy, and amplify the capacity to treat a larger number of cancer patients.

## Conclusions

The impact of AI-based autosegmentation of OARs in LMICs shows promise but comes with important caveats. Although AI software tools can assist in generating contours for OARs, the accuracy and reliability of these tools are not absolute and require further validation through prospective studies. Human oversight remains crucial to ensure accuracy and to integrate clinical judgment in the delineation of OARs. Additionally, the implementation of AI-based autosegmentation of OARs in LMICs faces significant challenges, including costs, infrastructure needs, internet connectivity, and training of radiation therapy staff. Addressing these challenges and limitations is essential for AI to significantly contribute to the rapid deployment of high-quality radiation therapy services and improve cancer care in LMICs. Further research is needed to optimize AI algorithms/models using patient data from LMICs. By leveraging AI technology, we can enhance access to high-quality radiation therapy services, ultimately contributing to improved cancer care in these regions.

## Disclosures

None.

## References

[1] Rahib L, Wehner MR, Matrisian LM, Nead KT. (2021). Estimated projection of US cancer incidence and death to 2040. JAMA Netw Open.

[2] Farmer P, Frenk J, Knaul FM (2010). Expansion of cancer care and control in countries of low and middle income: a call to action. Lancet.

[3] Omotoso O, Teibo JO, Atiba FA (2023). Addressing cancer care inequities in sub-Saharan Africa: current challenges and proposed solutions. Int J Equity Health.

[4] Elmore SN, Polo A, Bourque JM (2021). Radiotherapy resources in Africa: an International Atomic Energy Agency update and analysis of projected needs. Lancet Oncol.

[5] Datta NR, Samiei M, Bodis S. (2014). Radiation therapy infrastructure and human resources in low- and middle-income countries: present status and projections for 2020. Int J Radiat Oncol Biol Phys.

[6] Netherton TJ, Cardenas CE, Rhee DJ, Court LE, Beadle BM. (2021). The emergence of artificial intelligence within radiation oncology treatment planning. Oncology.

[7] Rattan R, Kataria T, Banerjee S (2019). Artificial intelligence in oncology, its scope and future prospects with specific reference to radiation oncology. BJR Open.

[8] Siddique S, Chow JCL. (2020). Artificial intelligence in radiotherapy. Rep Pract Oncol Radiother.

[9] Santoro M, Strolin S, Paolani G (2022). Recent applications of artificial intelligence in radiotherapy: where we are and beyond. Appl Sci.

[10] IAEA (2015).

[11] Kim F. Faculty Scholar expands project to provide advanced radiation therapy in Mongolia Global Health Center: Institute of Public Health, Washington University in St Louis; 2022.https://publichealth.wustl.edu/faculty-scholar-expands-project-to-provide-advanced-radiation-therapy-in-mongolia/.

[12] Brouwer CL, Steenbakkers RJ, Bourhis J (2015). CT-based delineation of organs at risk in the head and neck region: DAHANCA, EORTC, GORTEC, HKNPCSG, NCIC CTG, NCRI, NRG Oncology and TROG consensus guidelines. Radiother Oncol.

[13] Gay HA, Barthold HJ, O'Meara E (2012). Pelvic normal tissue contouring guidelines for radiation therapy: a Radiation Therapy Oncology Group consensus panel atlas. Int J Radiat Oncol Biol Phys.

[14] Duan J, Bernard M, Downes L (2022). Evaluating the clinical acceptability of deep learning contours of prostate and organs-at-risk in an automated prostate treatment planning process. Med Phys.

[15] Dice LR. (1945). Measures of the amount of ecologic association between species. Ecology.

[16] Liu Y, Lei Y, Fu Y (2020). Head and neck multi-organ auto-segmentation on CT images aided by synthetic MRI. Med Phys.

[17] Zhang Z, Zhao T, Gay H, Zhang W, Sun B. (2021). Weaving attention U-net: a novel hybrid CNN and attention-based method for organs-at-risk segmentation in head and neck CT images. Med Phys.

[18] Radici L, Ferrario S, Borca VC (2022). Implementation of a commercial deep learning-based auto segmentation software in radiotherapy: evaluation of effectiveness and impact on workflow. Life (Basel).

[19] Aoyama T, Shimizu H, Kitagawa T (2021). Comparison of atlas-based auto-segmentation accuracy for radiotherapy in prostate cancer. Phys Imaging Radiat Oncol.

[20] Greenham S, Dean J, Fu CK (2014). Evaluation of atlas-based auto-segmentation software in prostate cancer patients. J Med Radiat Sci.

[21] Urago Y, Okamoto H, Kaneda T (2021). Evaluation of auto-segmentation accuracy of cloud-based artificial intelligence and atlas-based models. Radiat Oncol.

[22] Strolin S, Santoro M, Paolani G (2023). How smart is artificial intelligence in organs delineation? Testing a CE and FDA-approved Deep-Learning tool using multiple expert contours delineated on planning CT images. Front Oncol.

[23] Manson EN, Hasford F, Trauernicht C (2023). Africa's readiness for artificial intelligence in clinical radiotherapy delivery: Medical physicists to lead the way. Phys Med.

[24] Zhao Y, Li H, Wan S (2019). Knowledge-aided convolutional neural network for small organ segmentation. IEEE J Biomed Health Inform.

[25] Amjad A, Xu J, Thill D (2022). General and custom deep learning autosegmentation models for organs in head and neck, abdomen, and male pelvis. Med Phys.

[26] van der Veen J, Gulyban A, Willems S, Maes F, Nuyts S. (2021). Interobserver variability in organ at risk delineation in head and neck cancer. Radiat Oncol.

[27] Savenije MHF, Maspero M, Sikkes GG (2020). Clinical implementation of MRI-based organs-at-risk auto-segmentation with convolutional networks for prostate radiotherapy. Radiat Oncol.

